# Temperature Dependent Effects of Elevated CO_2_ on Shell Composition and Mechanical Properties of *Hydroides elegans*: Insights from a Multiple Stressor Experiment

**DOI:** 10.1371/journal.pone.0078945

**Published:** 2013-11-12

**Authors:** Vera B. S. Chan, Vengatesen Thiyagarajan, Xing Wen Lu, Tong Zhang, Kaimin Shih

**Affiliations:** 1 Swire Institute of Marine Sciences and School of Biological Sciences, The University of Hong Kong, Hong Kong SAR; 2 Department of Civil Engineering, The University of Hong Kong, Hong Kong SAR; University of Connecticut, United States of America

## Abstract

The majority of marine benthic invertebrates protect themselves from predators by producing calcareous tubes or shells that have remarkable mechanical strength. An elevation of CO_2_ or a decrease in pH in the environment can reduce intracellular pH at the site of calcification and thus interfere with animal’s ability to accrete CaCO_3_. In nature, decreased pH in combination with stressors associated with climate change may result in the animal producing severely damaged and mechanically weak tubes. This study investigated how the interaction of environmental drivers affects production of calcareous tubes by the serpulid tubeworm, *Hydroides elegans*. In a factorial manipulative experiment, we analyzed the effects of pH (8.1 and 7.8), salinity (34 and 27‰), and temperature (23°C and 29°C) on the biomineral composition, ultrastructure and mechanical properties of the tubes. At an elevated temperature of 29°C, the tube calcite/aragonite ratio and Mg/Ca ratio were both increased, the Sr/Ca ratio was decreased, and the amorphous CaCO_3_ content was reduced. Notably, at elevated temperature with decreased pH and reduced salinity, the constructed tubes had a more compact ultrastructure with enhanced hardness and elasticity compared to decreased pH at ambient temperature. Thus, elevated temperature rescued the decreased pH-induced tube impairments. This indicates that tubeworms are likely to thrive in early subtropical summer climate. In the context of climate change, tubeworms could be resilient to the projected near-future decreased pH or salinity as long as surface seawater temperature rise at least by 4°C.

## Introduction

Marine benthic invertebrates protect their soft tissues from predators, pathogens, and abrasions with a tube or shell made of calcium carbonate (CaCO_3_) built using a sophisticated biomineralization process [1], [2], [3]. The biologically produced tubes typically have superior mechanical properties compared to naturally occurring inorganic CaCO_3_ [4], [5], [6]. These mechanical properties depend on the size and arrangement of the crystal units, the polymorph composition (calcite/aragonite ratio), the degree of calcium substitution in the CaCO_3_ mineral lattice by magnesium (Mg) and strontium (Sr), and the quantity and quality of the organic matrix [7], [8]. The growth of the crystals, in terms of its shape and structure, is partially determined by environmental pH, salinity, and temperature [9], [10], [11]. For example, the Mg/Ca ratio in shells linearly increases with increasing environmental temperature and can thus be used as a paleothermometer [12]. In addition, the precipitation of aragonite with respect to calcite increases with temperature [13]. Similarly, the shell Sr/Ca ratio increases with increasing salinity [14]. Environmental pH is one of the key determining factors for CaCO_3_ crystallization that can affect marine organisms externally by reducing the CaCO_3_ mineral saturation state and internally by reducing physiological pH [15], [16]. Decreased pH in the environment and changes in the carbonate system caused by natural or anthropogenic processes can severely alter the composition and mechanical properties of the shells [17], [18]. However, how climate change stressors interact and affect tube or skeletal structures is not well-understood and studies in this area are lacking [19], [20], [21], [22].

Much like molluscs and corals and other marine invertebrates, the serpulid tubeworms construct calcareous tubes by a complex biomineralization process [23], [24]. To protect the animals against predators, the serpulids commonly produce calcareous tube with a variety of ultrastructures [25]. In addition, some serpulids can also construct a calcified operculum [26]. The tubeworms can build calcareous reef structures that become physical habitats and refuges for a variety of benthic animals [27]. On the other hand, massive accumulation of calcareous reefs on man-made marine installations such as ship hulls, known as biofouling, leads to severe economic loss for marine industries [28]. In subtropical coastal waters of Hong Kong, the tubeworm population typically peaks in winter conditions, i.e. lower temperature (∼23°C), high salinity (∼34‰) and high pH (∼8.1) [29]. The tubeworm population declines rapidly as the temperature rises (∼29°C) and salinity drops (∼27 ‰ in eastern waters) in the summer wet season, most likely due to the narrow range of tolerance to the summer conditions [29]. Recent increases in anthropogenic CO_2_ has decreased the pH (from 8.2 to 8.1) in estuarine and shallow coastal areas, and together with the predicted elevation of CO_2_ in the near-future (with pH 7.6−7.8) threaten the survival of this ecologically and commercially important tubeworm species [30]. The sea surface temperature and precipitation are also predicted to rise in the future. Therefore, the effects of elevated CO_2_ together with elevated temperature and freshening of seawater in early spring may interact to limit the *H. elegans* population in the subtropical waters by narrowing the environmental window suitable for the production of calcareous tubes.

Despite much concerted effort to understand the process of biomineralization in a variety of marine organisms [31], [32], [33], including tubeworms [34], [35], [36], the effect of environmentally and climatically relevant stressors on the composition and mechanical function of tubes or shells has yet to be fully studied. Specifically, no studies have been done on how the interaction of multiple environmental variables affects calcareous structures and their mechanical properties, which could have many implications such as the susceptibility to predators. In this study, we investigated how environmentally realistic sub-lethal levels of stressors altered the production of calcareous tubes of the tubeworm, *H. elegans*. Specifically, we evaluated the effect of different combinations of environmental stressors on the accreted tubes in terms of biomineral composition, ultrastructure and overall mechanical properties.

## Materials and Methods

### Ethics Statement

No special permits were required for the described field site and sampling. No specific permissions were required for tubeworm sample collection from Yung Shue O, Hong Kong in the South China Sea. We also confirm that the location is not privately-owned or protected in any way and the field studies did not involve endangered or protected species.

### Collection and Maintenance of Test Animal

In April 2012, serpulid tubeworms, *Hydroides elegans*, were collected from aggregates on floating structures in a fish farm in Yung Shue O, Hong Kong (22°27′N, 114°23′W). The conditions at the collection site, salinity (∼34 ‰), surface water temperature (∼23°C) and pH (∼8.1) were noted. The collected tubeworms were transported in seawater to the laboratory and acclimatized for 2 to 3 days in an outdoor tank with running seawater at ambient conditions. Gametes were obtained from more than 100 individuals by gently breaking open their tubes [37]. Eggs were incubated in 0.22 µm filtered seawater (FSW; 34‰ salinity, pH 8.1) with an appropriate amount of sperm [38]. After 2 hours, the majority of eggs had been fertilized and the early embryos were separated from the sperm for use in the following experiment.

### Experimental Design

Using an orthogonal factorial experimental design, we tested the individual and combined effects of pH, salinity and temperature on the composition, mechanical properties and ultrastructure of the calcareous tube constructed by the 3-week-old *H. elegans* adults. For the factorial manipulation experiments, each independent factor had two levels: pH 8.1 (ambient or control) and pH 7.8, salinity of 34‰ (ambient) and 27 ‰, and temperature of 23°C (ambient) and 29°C. In total, there were eight water treatment combinations with four replicates for each treatment. The selected temperature and salinity levels were within the ranges experienced by tubeworms in the natural habitat in the subtidal region of Hong Kong waters [29]. Ambient salinities in the tubeworm habitat may drop to as low as 15‰ during late spring months (April to May) due to early monsoon rain [37]. At the same time, surface seawater temperature can fluctuate between 18°C to 28°C during the sharp monsoon transition periods. The pH values in Hong Kong coastal waters naturally vary between 8.2 and 7.8 (*p*CO_2_ 500 to 1000 µatm) [39], [40]. Furthermore, the surface temperature of seawater is predicted to increase by 4°C within this century. Meanwhile, the pH is estimated to decrease by 0.35 units due to regional and global climate change and to rising anthropogenic atmospheric CO_2_ [41]. Although there is no salinity projection data for Hong Kong coastal waters, the experimental salinity level of 27 ‰ was chosen to avoid the lethal low salinity level of 25‰ [29]. All the experimental treatment values were chosen to reproduce environmentally realistic levels in the naturally fluctuating coastal waters of Hong Kong.

### Water Treatment Maintenance and Monitoring Procedures

The required temperature in the circulating water bath was maintained using an immersed water heater. The salinity of ambient FSW was lowered from 34 to 27‰ by dilution with MilliQ distilled water [37]. The decreased pH was obtained by bubbling CO_2_ enriched air into treatment tanks [42] and the flow rate of CO_2_ and air was adjusted using a variable area flow meter controller (Cole-Parmer Inc., USA) [43]. The ambient pH 8.1 was decreased to about pH 7.8 by CO_2_ maintained at about 1000 ppm, which was fixed because CO_2_-driven changes in the pH and carbonate system are dependent on seawater temperature and salinity. The CO_2_ concentration in the inflow air was frequently measured using a Quantek Model 906 Carbon Dioxide Analyzer (Quantek Instruments, Inc., USA). The CO_2_ analyzer was calibrated using a gas standard (Hong Kong Oxygen & Acetylene Company Limited, Hong Kong).

Temperature and pH were monitored daily using a pH meter (SG2, Mettler-Toledo, Hong Kong) calibrated using NBS/NIST standards (pH 4, 7 and 10). Salinity was measured daily using a refractometer. For the total alkalinity (TA) measurements, seawater samples (50 mL) were poisoned with 10 µl of 250 mM mercuric chloride and TA was measured using an Alkalinity Titrator (Apollo SciTech Inc., USA). The measurement procedure was validated using the certified seawater reference materials (Batch 103, A.G. Dickson, Scripps Institution of Oceanography) [44]. The carbonate system parameters in each experimental unit were obtained using the CO2SYS program with the equilibrium constants K1, K2 and KSO4 [45].

### Exposure of Test Animals to the Treatments and Sample Collection

Embryos of *H. elegans* were exposed to each of the different treatments and were mass reared in 1 L plastic culture beakers (10 embryos per ml) for about 3 weeks (21 to 22 days) until the adult stage. Culture beakers were capped to prevent changes in salinity and to reduce entry of ambient air. Cultures were maintained under fluorescent room lights and at the corresponding treatment conditions using the procedures described above. Once every 2 days, the entire culture medium was replenished and concentrated algal (*Isochrysis galbana*) suspensions (at about 10^5^ cells per ml) were added as a food source for the larvae or adults [29]. The culture medium was changed frequently to allow larvae or adults to feed *ad libitum* to promote active growth, and to minimize the effects of algae on the seawater carbonate chemistry [43]. Under these culture conditions, embryos reached competency to attach and metamorphose into juvenile tubeworms after 4 and 5 days for the 29°C and 23°C treatments, respectively. Larvae were induced to metamorphose on plastic petri dishes coated with natural biofilm (petri dishes had been treated in flowing natural seawater for 7 days) [30]. Within 24 hours, the majority of larvae (>70%) had metamorphosed. Unattached larvae were removed and attached juveniles were allowed to grow. Post-metamorphic tubeworms were continuously exposed to their respective treatment conditions for 17 days after metamorphosis. At the end of the experiment, adult tubeworms were imaged under a microscope coupled with a digital camera (Leica DFC 280, Germany). An average of eight randomly selected individuals per replicate had their tube lengths measured using image analysis software (ImageJ version 1.45s, USA). Adult tubeworms were rinsed in MilliQ water and killed with 70% ethanol. Tubes were air dried and kept attached to petri dishes at room temperature for the subsequent analysis of their composition, mechanical strength and ultrastructure.

### Analyses of Tube Composition

The combined effect of pH, salinity and temperature on tube mineral composition was assessed using X-ray powder diffraction (XRD), Fourier transform infrared spectrometry (FTIR) and inductively coupled plasma optical emission spectrometry (ICP-OES). Composition of tubes in terms of calcite/aragonite ratio and relative amorphous calcium carbonate (ACC) content were quantitatively analyzed using XRD and FTIR, respectively. About 8–10 randomly selected tube samples were dislodged from the petri dish (per replicate) and cleaned to remove the organic soft tissues using 5% bleach (NaOCl, Clorox ™) for about 30 minutes [34]. The tubes were then rinsed twice with double distilled water, air-dried, and ground into fine powder and stored in weighing paper sachets for XRD and FTIR analyses. Powder samples from 8–10 tubeworms were digested using 2% nitric acid for ICP-OES [46].

The X-ray diffraction pattern for each sample was obtained using a Bruker D8 Advanced X-ray powder diffractometer equipped with a Cu Kα radiation source and a LynxEye detector [43]. The system was calibrated using a Standard Reference Material 660a (lanthanum hexaboride, LaB_6_, U. S. National Institute of Standard and Technology, USA). The diffractometer parameters were 40 kV, 40 mA, a 2*θ* scan range of 10° to 110°, step size of 0.02° and a scan speed of 0.3 s/step. Mineral phases were identified and quantified using Eva XRD Pattern Processing software (Bruker, USA). Powder XRD patterns were matched with the powder diffraction standards database of the International Centre for Diffraction Data (ICDD PDF-2 Release 2008). The Rietveld refinement method for quantitative analysis of the phase compositions was processed by the TOPAS (version 4.0) crystallographic program [47].

For FTIR analysis, tube powder samples (∼1 mg) were mixed with KBr (∼10 mg; KBr was dehydrated at 98°C overnight) and compressed under a pressure of 9 tons for 2 minutes to produce a pellet (diameter, 13 mm). Infrared absorption spectra were obtained using FTIR (L120–000B, Perkin Elmer, USA) in the range 500–2000 cm^−1^ with 1 cm^−1^ resolution. Infrared spectra were analyzed with Spectrum One software (version 3.02) and after baseline removal, the peak heights at 855 cm^−1^ (*ν*_2_) and 713 cm^−1^ (*ν*_4_) corresponding to internal vibration modes of CO_3_^2-^ ions were measured [48]. Magnesium/calcium (Mg/Ca) and strontium/calcium (Sr/Ca) ratios in the tube samples were determined using ICP-OES. Three dilutions of the acid digested analytes were prepared and analyzed independently (1-fold, 10-fold, 100-fold dilutions). Elemental ratios were determined from the relative intensities measured for calcium (396.847 nm), magnesium (285.213 nm) and strontium (407.771 nm) using ICP-OES (PE Optima 8300, Perkin Elmer, USA). The ratios were calculated from the respective dilutions to ensure they were quantified within the linear calibration ranges [49].

### Analysis of Tube Ultrastructure

Analysis of the tube ultrastructure was performed with a scanning electron microscopy (SEM) [36]. The tubes were embedded in resin and were sectioned perpendicular to the longitudinal axis of the tube. The cross-sectional surfaces were smoothed by sectioning with a diamond knife in an ultramicrotome (Ultracut S, Leica, Germany). The specimens were etched for 1 minute in 0.5 M EDTA solution to reveal the tube ultrastructure [23]. Tube sections were mounted face up onto aluminum stubs with carbon tape. The surrounding resin surfaces were painted with silver to minimize electron charging, and the sectional surfaces were sputter coated with 50 nm of a gold-palladium alloy. SEM microscopic images were taken using a Leo 1530 FEG SEM (Zeiss LEO, Germany). Tube thickness was also measured from the SEM images.

### Analysis of Tube Mechanical Properties

The effects of the different treatments on tube hardness (H) and Young modulus of elasticity (E) were determined using nanoindentation apparatus [6], [50], [51]. After the SEM imaging analysis, the gold-palladium alloy coating together with the etched layers of sectional surfaces were removed by further sectioning using the ultramicrotome with a diamond knife. The sectioned specimen showed a smooth surface with minimal topography and was suitable for nanoindentation tests [52]. The specimens were mounted face up and tested under dry ambient conditions with a triboindenter (Hysitron Inc., USA) equipped with an in-situ scanning probe microscope (SPM). Tube samples from the treatment group pH 7.8, 27 ‰ and 23°C were not analyzable for nanoindentation measurement due to insufficient tube thickness (∼6–7 µm) and loss of a few fragile tube samples during embedding procedures. Four to nine measurements from random but sufficiently spaced locations were taken from each specimen [43]. Measurements from the surfaces at the bottom of the tube were excluded to minimize discrepancies in the analysis, because these adhering surfaces have distinctive mineralogy and structures. A Quartz Berkovich three-sided pyramid indentor was monitored at a load resolution of <1 nN and a displacement resolution of 0.1 nm. Specimens were indented with controlled peak loads between 1500 and 7000 µN to achieve sufficient depth of penetration from 141.1 to 568.4 nm, which was adequate for determining the contact area between the indenter and the specimen [4], [53]. The load function can be divided into three segments: a standard 5 sec load phase; 3 sec hold; and the unload phase, where the load decreases at the same rate as the load phase until reaching zero load. Tube mechanical properties, H and E, were obtained from the recorded load-displacement curves for each recorded measurement.

### Statistical Analysis

The effects of the interaction of three environmental factors, temperature, salinity and pH, on the tube characteristics were analyzed using three-way ANOVA. Data were transformed (square root or log) to achieve normality and equality of variance for ANOVA. Because of natural and inherent variability within and among treatments, the power of three-way ANOVAs were generally low to eliminate the likelihood that treatment effects were due to random sampling variability [54]. To overcome this issue, the Student’s t-test was used to compare the treatment effects with the control. Since Bonferroni correction for multiple comparisons reduces type I errors but increases type II errors, we did not adjust *p* values but reported probabilities and interpreted data with caution based on differences between the magnitude of means [54]. The significant results in three-way ANOVA table which were indicative of strong treatment effects were analyzed. Tube composition and structural data were also analyzed by principal component analysis. The relationship between treatment variable and tube composition or between tube hardness and elasticity were analyzed using Spearman rank correlation.

## Results

### Effect of CO_2_ (pH), Salinity, and Temperature on the Carbonate System

**Figure 1:**
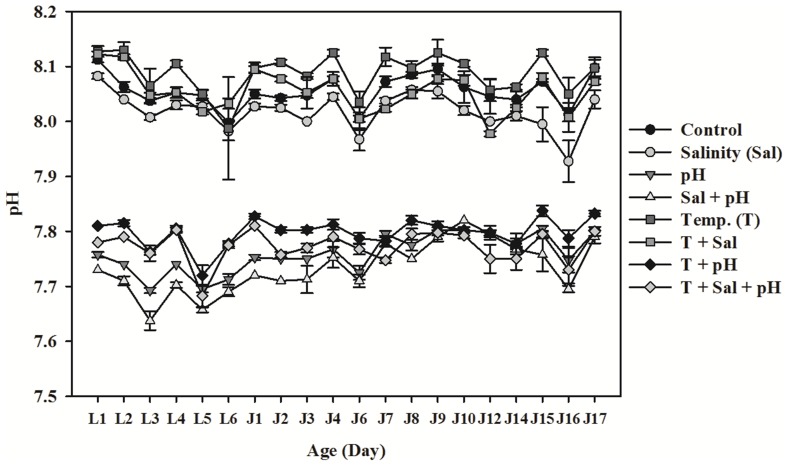
Mean pH (NBS scale) change during the experiment. The mean ± SD of four replicates. Abbreviations are L1–L6: Day 1–6 of larval growth phase; J1–J17: Day 1–17 of post-settlement juvenile/adult growth phase; Control (pH 8.1, Salinity 34‰ Temperature 23°C); Salinity (pH 8.1, Salinity 27‰ and Temperature 23°C); Temperature (pH 8.1, Salinity 34‰ and Temperature 29°C); pH×Sal: 7.8 and 27; pH×T: 7.8 and 29; Sal×T: 27×29; pH×Sal×T: 7.8×27×29.

The monitored pH, salinity, and temperature values throughout the experiments did not deviate much from our experimental values (Figure 1). Similarly, the fixed variables did not fluctuate much among replicate cultures for any given treatment condition (Table 1). A steady concentration of CO_2_ (∼1000 µatm) in the bubbled air reduced the pH from 8.1 to ∼7.8 in all the decreased pH treatments except the treatment involving elevated temperature and reduced salinity, where the pH was decreased to ∼7.75 (Table 1). As expected, reduced salinity decreased both the CO_3_^2−^ concentration and calcite saturation level by decreasing Ca^2+^ ion concentration, but this did not affect pH or *p*CO_2_ level. At ambient pH and salinity, elevated temperature significantly increased the aragonite saturation level (Ω_A_) from 2 to 2.6. At decreased pH, the Ω_A_ was 1.3, but was reduced to 1.0 in decreased pH and reduced salinity. On the other hand, the elevated temperature raised the Ω_A_ to 1.6 when combined with reduced salinity or decreased pH. However, with all three factors combined (i.e. T +Sal+pH), the Ω_A_ was 1.2 (Table 1). The dissolved oxygen (DO) levels were relatively constant across eight treatment manipulations (Table 1). This stable and controlled experimental system allowed us to quantify the effects of multiple environmental stressors on calcareous tube composition, mechanical strength and structure.

**Table 1:** Measured and calculated values (mean ± SD; n = 4) of carbonate system parameters from a representative day, e.g. 12 day post-settlement or J12 in Figure 1.

	Measured parameters					Calculated parameters			
Treatment	pH (NBS scale)	Salinity (‰)	Temp (°C)	TA (µmol kg^−1^)	DO (mg l^−1^)	*p*CO_2_ (µatm)	CO_3_^2-^ (µmol kg^−1^)	Ω_calcite_	Ω_aragonite_
Control	8.05±0.03	34.0±0.1	22.3±0.1	2033±254	5.01±0.27	516±44	131±7	3.1±0.18	2.1±0.12
Salinity (Sal)	8.00±0.01	27.0±0.1	22.3±0.1	1623±129	5.71±0.06	495±01	82±0.1	2.1±0.01	1.3±0.01
pH	7.80±0.01	34.0±0.1	22.4±0.2	2044±480	4.87±0.27	985±14	80±0.5	1.9±0.01	1.3±0.01
Sal+pH	7.80±0.01	27.0±0.1	22.5±0.1	1753±670	5.11±0.49	904±13	58±0.6	1.4±0.01	1.0±0.01
Temp. (T)	8.06±0.02	34.0±0.1	22.5±0.1	1753±670	5.11±0.49	904±13	58±0.6	1.4±0.01	1.0±0.01
T+Sal	7.98±0.01	27.0±0.1	29.4±0.1	1633±250	5.14±0.21	550±07	96±0.9	2.5±0.02	1.6±0.01
T+ pH	7.80±0.01	34.0±0.1	29.4±0.1	1990±145	5.28±0.16	1005±33	95±2.4	2.3±0.05	1.6±0.03
T+Sal+pH	7.75±0.03	27.0±0.1	29.4±0.1	1884±166	5.26±0.14	1147±74	70±3.8	1.8±0.09	1.2±0.06

DO, dissolved oxygen level; CO_3_^2-^, carbonate ion; TA, total alkalinity of seawater; *p*CO_2_, partial pressure of CO_2_; Ω_calcite_, calcite saturation level; Ω_aragonite_, aragonite saturation level.

### Tube Size

The effect of treatments on the tube size was measured in terms of total length and thickness. Among the three factors, only temperature had a significant positive effect on tube length (Table S1). After 17 days post-settlement growth (excluding the 4 or 5 days of larval growth before settlement for 29°C and 23°C, respectively), the tube length ranged from 3.5 to 5.0 mm at 23°C and from 6.0 to 7.0 mm at 29°C. However, the three factors either individually or in combination did not affect tube thickness, which varied between 8 to 20 µm.

### Tube Composition

**Figure 2:**
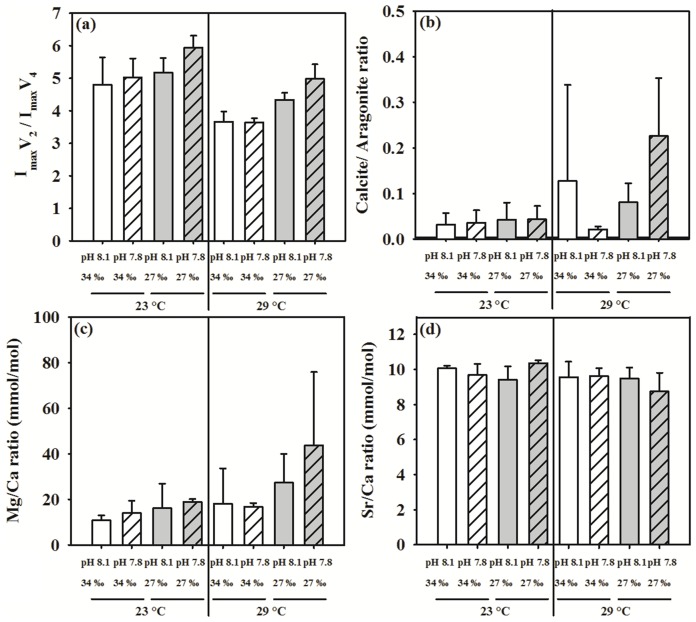
Effects of pH, salinity and temperature on the tube composition of *Hydroides elegans*, (a) the intensity ratio (I_max_) of the two FT-IR absorption peaks at 855 cm^−1^ (*ν*_2_) and 713 cm^−1^ (*ν*_4_), I_max_ *ν*_2_/I_max_ *ν*_4_ ratio, is an indicator of amorphous CaCO_3_ content; (b) calcite/aragonite ratio, (c) Mg/Ca ratio, (d) Sr/Ca ratio. Each bar represents the mean ± SD of four replicates.

Elevated temperature significantly decreased the ACC content of the tubes (Figure 2a, Table 2). According to the mean square (MS) values in the ANOVA table (Table S2), the magnitude of the effect of temperature on ACC content was twice as significant as the effect of pH, and was much higher than the other insignificant single or interacting factors. When isolated from ANOVA testing, elevated temperature alone or in combination with decreased pH 7.8 dramatically reduced the ACC content (Student’s t-test (s), *p*<0.01; right panel in Figure 2a). Both the calcite/aragonite ratio and the Mg/Ca ratio increased in response to elevated temperature regardless of decreased pH and reduced salinity (Figure 2b and c). Noticeably, these two ratios increased by about 3-fold in response to the combined effect of the three stressors (Figure 2b and c; Table 2; Tables S2, S3). In contrast, the tube Sr/Ca ratio showed more complex factor interactions (pH×Salinity×Temperature, *p*<0.05; Figure 2d, Table 2, Table S3). The Sr/Ca ratio was shown to be dependent on all three stressors combined, unlike the other examined tube compositions. For example, reduced salinity led to a reduction in Sr/Ca ratio at 29°C, but not at 23°C (Student’s t-test, *p*<0.01).

**Table 2:** Summary results of 3-way ANOVA showing the *F*-ratios for the main and interactive effects of pH (8.1 and 7.8), salinity (27 and 34 ‰) and temperature (23°C and 29°C) on (1) tube composition in terms of amorphous CaCO_3_, calcite/aragonite ratio, Mg/Ca ratio, Sr/Ca ratio, and (2) mechanical properties, in terms of hardness and elasticity.

	pH	Sal	T	pH×Sal	pH×T	Sal×T	pH×Sal×T
Tube composition							
Amorphous CaCO_3_	**20.865**	1.593	**41.841**	3.553	1.223	0.239	0.001
Calcite/aragonite ratio	**6.144**	0.107	**4.557**	1.615	3.19	0.001	2.148
Mg/Ca ratio*	5.393	1.116	5.293	0.744	1.714	0.208	0.821
Sr/Ca ratio	1.019	0.017	**5.164**	0.32	1.123	1.782	**5.18**
**Tube mechanical properties**							
Hardness	1.937	2.794	**5.597**	0.197	0.963	1.693	0.01
Elasticity	1.329	1.891	**5.683**	0.018	0.774	1.552	0.144

Significant effects (*p*<0.05) are indicated in bold. Detailed information of ANOVA results are provided in tables S1 to S3. pH×Sal: 7.8 and 27, pH×T: 7.8 and 29; Sal×T: 27×29; pH×Sal×T: 7.8×27×29.

* As Mg/Ca ratio did not fulfill requirement of variance homogeneity, the critical p-value was adjusted to a more conservative value of p<0.01.

### Tube Mechanical Properties

**Figure 3:**
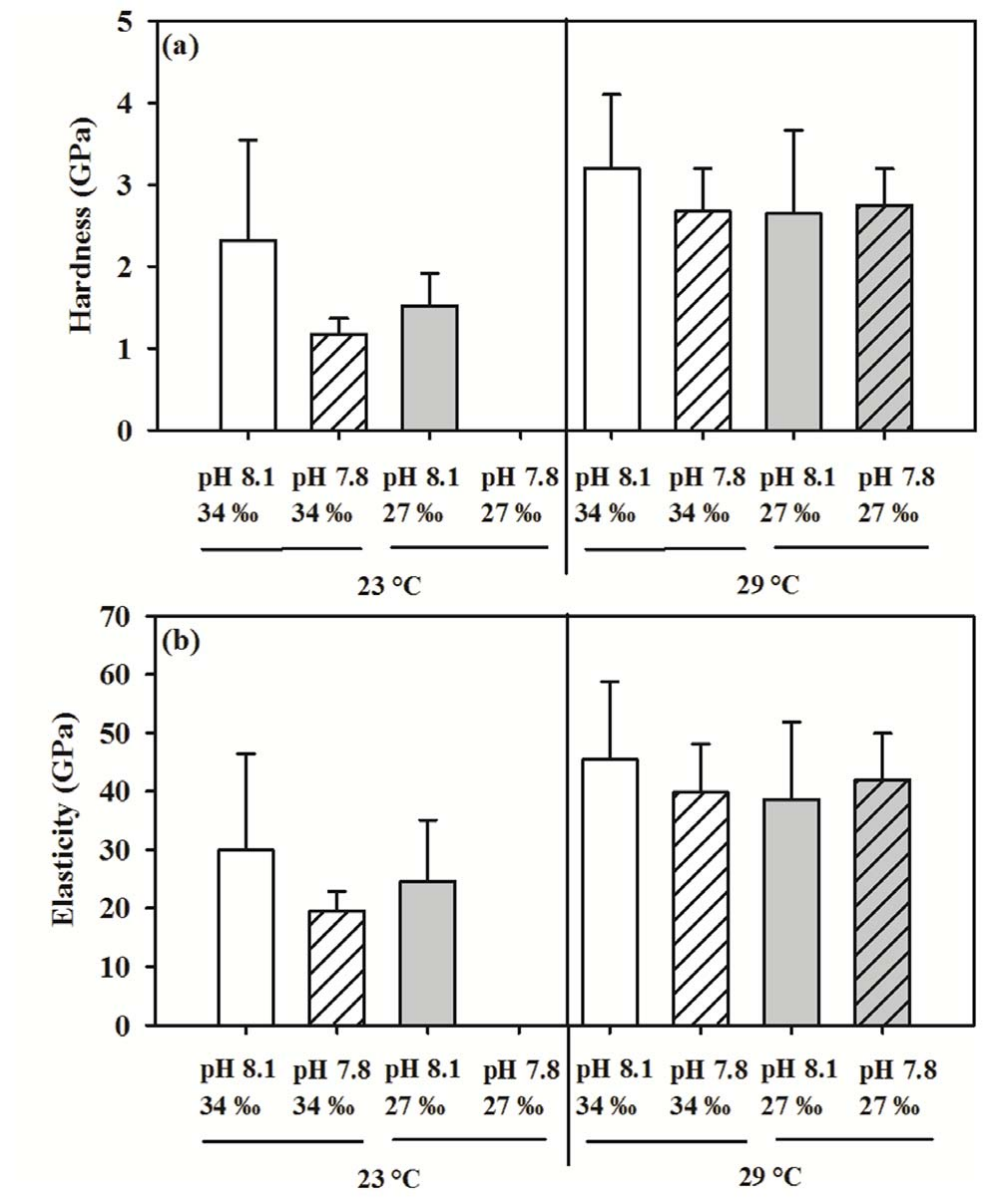
Effects of pH, salinity and temperature on mechanical properties of the tubes built by *Hydroides elegans*, (a) hardness and (b) elasticity. Each bar represents the mean ± SD of four replicates, except control had four replicates.

**Figure 4:**
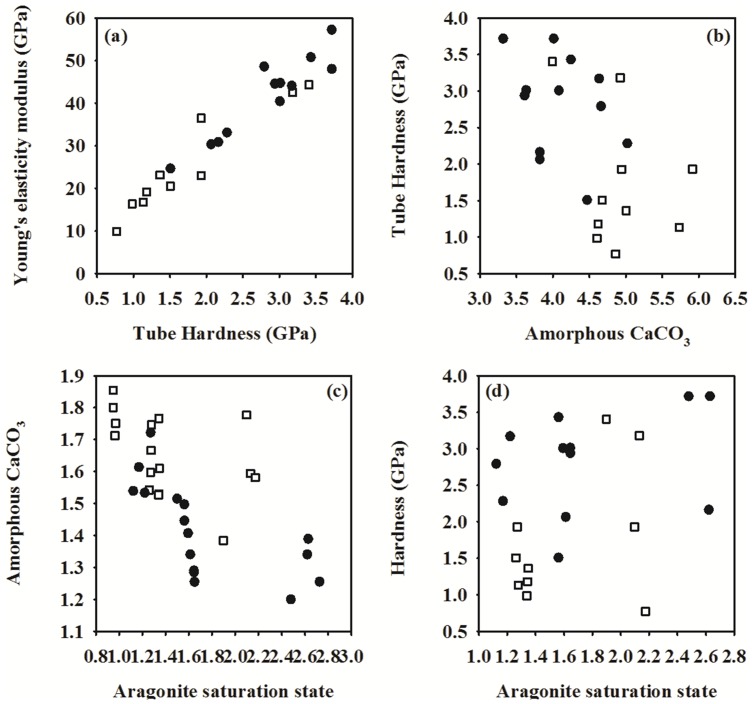
The relationship between (a) tube hardness and elasticity, (b) tube ACC and tube hardness in the *Hydroides elegans*. (c) tube ACC and aragonite saturation level, and (d) between tube hardness and aragonite saturation level. Solid circles represent samples obtained from all the four elevated water temperature (29°C) treatments. Empty squares represent samples obtained from all the four ambient water temperature (23°C) treatments.

The three-way ANOVA revealed that the temperature had a significant effect on the measured tube mechanical properties (Figure 3a, b; Table 2). Elevated temperature had a greater positive effect on tube hardness and elasticity and rescued the effects caused by decreased pH and reduced salinity (Student’s t-tests, *p*<0.01). Regardless of treatment conditions, a positive linear correlation was observed between tube hardness and elasticity (Spearman correlation r_s_ = 0.91, *p*<0.001; Figure 4a). Notably, the hardness increased with decreasing ACC (Spearman correlation r_s_ = −0.51, *p*<0.01; Figure 4b).

### Relationship between Carbonate System Variables and Tube Properties

**Figure 5:**
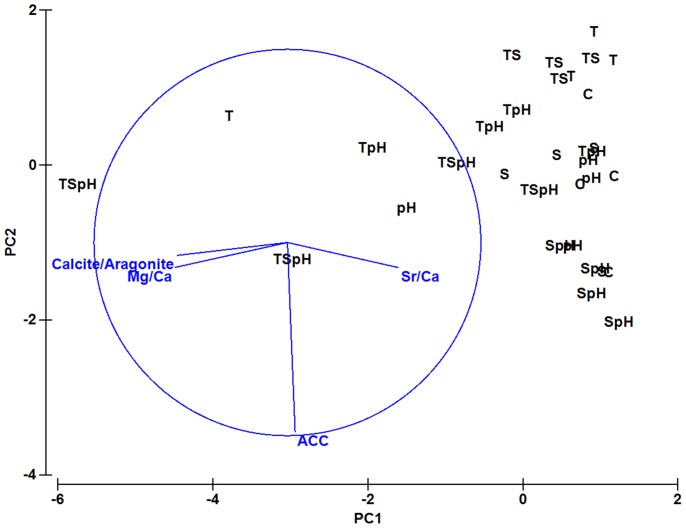
The principal component analysis (PCA) showing the global differences among tube samples collected from all the eight water treatment conditions (“C” = control treatment 23°C, pH 8.1, 34‰; “S” = low salinity treatment 23°C, pH 8.1, 27‰; “pH” = high CO_2_ treatment, 23°C, pH 7.8, 34‰; “SpH” = low salinity and high CO_2_ treatment, 23°C, pH 7.8, 27‰; “T” = warm temperature treatment, 29°C, pH 8.1, 34‰; “TS” = warm temperature and low salinity treatment 29°C, pH 8.1, 27‰; “TpH” = warm temperature and high CO_2_ treatment, 29°C, pH 7.8, 34‰; “TSpH” = warm temperature low salinity and high CO_2_ treatment, 29°C, pH 7.8, 27‰) in two different components, PC1 and PC2. A loading plot for the normalized data values is also shown on the PCA plot (circle).

The carbonate ion concentration and aragonite saturation state had a strong and significant correlation with the tube ACC content (Spearman correlation r_s_ = −0.68, *p*<0.05 Table 3, Figure 4c) as well as with the tube hardness (Spearman correlation r_s_ = 0.50, *p*<0.05; Table 3, Figure 4d). As the aragonite saturation level decreased, the ACC content increased and hardness decreased. The relationship between treatment groups and tube composition (ACC content, aragonite/calcite ratio, Mg/Ca and Sr/Ca ratios) was also analyzed using a principal component analysis. The first two principal components explained >70% of variance (Figure 5). Majority of elevated warm water treatments are grouped and placed on the left hand side of the PCA plot at PC1. Correlation analysis results reported in Table 3 and Figure 4 have been corroborated by PCA analysis, that is, ACC content with Sr/Ca ratio explained >50% variance in PC1.

**Table 3:** Spearman rank correlations (r_s_) between *Hydroides elegans* tube composition (tube length, tube thickness, calcite to aragonite ratio, amorphous CaCO_3_ (ACC), magnesium to calcium ratio (Mg/Ca) and strontium to calcium ratio (Sr/Ca) and seawater carbonate ion (CO_3_^2-^) concentration or aragonite saturation level; and between tube mechanical properties (tube hardness and elasticity modulus) and CO_3_^2-^ or saturation state.

		CO_3_^2-^		Aragonite saturation	
Response Variable	N	r_s_	*p*	r_s_	*p*
Calcite/Aragonite	31	0.31	0.08	−0.33	0.06
ACC	32	**0.68**	0.01	−**0.68**	0.01
Mg/Ca	31	0.32	0.07	−0.32	0.07
Sr/Ca	31	0.06	0.73	−0.07	0.71
Hardness	21	**0.51**	0.01	**0.50**	0.02
Elasticity	21	0.31	0.16	0.30	0.18

Significant (*p*<0.05) correlation coefficient values are shown in bold. To avoid the distorting effect of outliers, the correlation analysis was performed after the outlier was removed. The removed outlier values are circled in the Figure 5.

### Tube Ultrastructure

**Figure 6:**
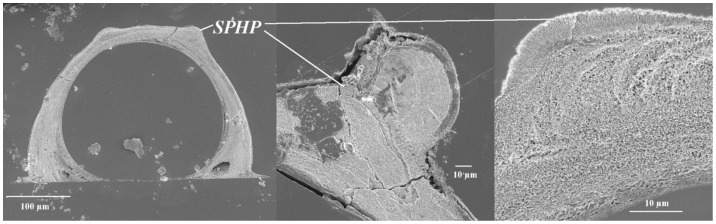
The SEM image showing a unique accretion of a spherulitic prismatic (SPHP) outer layer on a pair of “ridges” on tubes produced at elevated temperature (29°C) by the *Hydroides elegans*.

**Figure 7:**
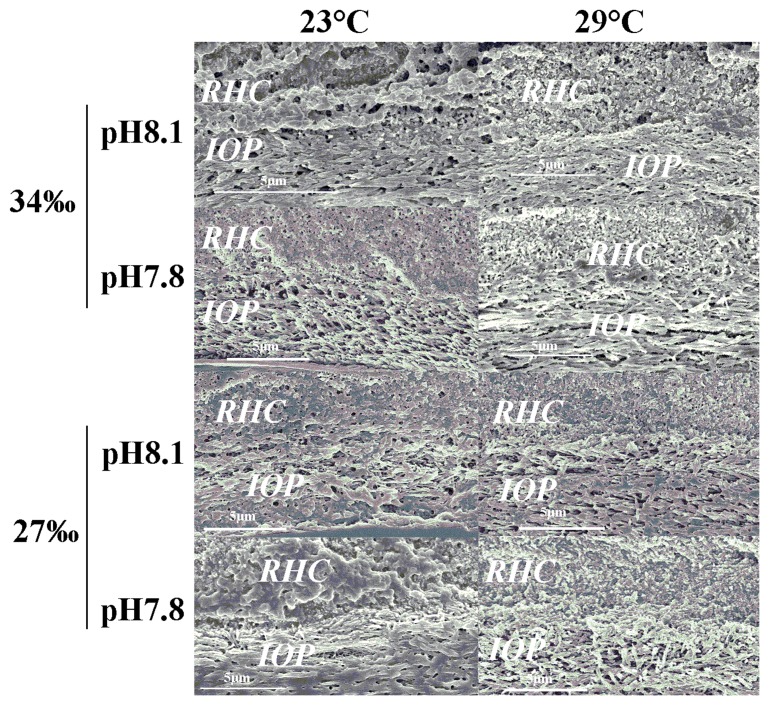
Effects of pH, salinity and temperature on cross sectional views of the tube ultrastructure in the *Hydroides elegans.* IOP are irregularly oriented prismatic structures and RHC are round homogenous crystal structure.

The outermost layer had a spherulitic prismatic structure (SPHP) and was prominent on tubes constructed in elevated temperature treatments regardless of changes in pH and salinity. Moreover, the two ridges observed at elevated temperature were not observed on tubes constructed at ambient temperature (Figure 6). Beneath the SPHP, we observed a middle layer composed of a rounded homogeneous crystal structure (RHC), and an innermost layer composed of an irregularly oriented prismatic structure (IOP). Comparative observation of the two layers of tube ultrastructure showed no obvious differences among treatments (Figure 7), except tubes produced at 29°C seemed to be more compact and less porous than those produced at 23°C.

## Discussion

This study used a factorial experimental design to investigate how the interaction of environmental stressors affected the composition, mechanical strength and structure of calcareous tubes of the reef building tubeworm, *Hydroides elegans*. Among the three climate change variables of decreased pH, reduced salinity and elevated temperature, we observed no significant synergistic or antagonistic effects on most of the examined tube features, except for the Sr/Ca ratio measurement. Tubeworms were able to produce tubes with well-defined ultrastructures even when exposed to all of the stressors individually or combined. Notably, at elevated temperature (29°C) with decreased pH and reduced salinity, the constructed tubes had similar values of hardness and elasticity as the control tubes, despite the negative effects caused by the reduced pH and salinity. The reduction in amorphous CaCO_3_ (ACC) content showed an interesting correlation with the overall mechanical properties of the tube. The combination of elevated temperature (29°C), reduced salinity (27 ‰) and decreased pH (7.8) is predicted to occur in the subtropical coastal areas within this century. Our results suggest that due to the rescuing effects of rising temperature, *H. elegans* would still be able to produce strengthened tubes with enhanced mechanical properties under these conditions. Implications of the observed environmental-mineral interaction, including the underlying mechanisms of specific effects of temperature, salinity and pH on tube composition, strength and structure are discussed below.

### Tube Composition: Effect of pH, Salinity and Temperature

This study highlighted three key insights into the interactions between environmental variables and calcareous tube composition. Using FTIR spectrometry, we found that tubes built at an elevated temperature of 29°C had significantly lower ACC content than those built at the ambient temperature of 23°C (Figure 2a; Table 2). The combination of ambient temperature with decreased pH and reduced salinity dramatically increased the ACC content, but combinations with elevated temperature had the antagonistic effect of reducing ACC content. The ACC is recognized as a transient, unstable, soluble precursor that is ultimately transformed into stable crystalline product such as calcite or aragonite [55], [56], [57]. The conversion of ACC to the crystalline forms may be more kinetically favorable at 29°C than at 23°C, possibly due to temperature-dependent physiological processes and elevated metabolic activity of organism. Nevertheless, low ACC content implies that tubes have a relatively stronger crystalline structure at 29°C than at 23°C.

Second, the calcite/aragonite ratio increased by about 3-fold in the elevated temperature treatments with the exception of the decreased pH treatment at 29°C. The precipitation of aragonite or calcite is determined by the Mg/Ca ratio in the seawater environment at the time of animal’s evolution [58], [59], [60]. The earliest serpulids appeared in the aragonitic seas of the Triassic era [61], this suggests that aragonite is the primitive biomineral for the calcareous tube. Apart from being influenced by the evolutionary history, the CaCO_3_ polymorph ratio also seems to have certain degree of plasticity to changes in the environment such as pH [62], [63], temperature [64] and salinity [14]. For instance, when the temperature was elevated from 5°C to 35°C, aragonite formation was favored at the warmer temperatures [65]. Serpulid tubeworms have evolved to have a sophisticated biomineralization mechanism and they control CaCO_3_ crystal production and assemblage in cellular compartments that are isolated from body fluids and from the outside environment [66]. Generally, animals have physiological mechanisms that maintain an optimal intracellular environment for mineralization regardless of the environmental conditions [67], [68], normal mineralization can be maintained if the animal can afford the extra energetic cost in response to suboptimal environments. For example, the response of *H. elegans* to the experimental conditions (29°C or 27‰ or pH 7.8) is associated with the ability of the animal to physiologically adjust. Faced with energy demanding circumstances, such as decreased pH, reduced salinity and elevated temperature, it could be more favorable to produce calcite, which has a lower density and typically contains less organic components than aragonite [69]. Mineral composition also depends on the animal’s ability to maintain skeletal integrity, particularly from dissolution under decreased environmental pH or other stressors such food limitation [70], [71]. However, this study did not examine the tube composition with respect to dissolution.

Third, when the results were pooled across all treatments, we found a significant negative correlation between the aragonite saturation state and ACC content of the tube. Consistent with previous findings [62], [63], aragonite saturation levels seemed to be one of the major driving forces of tube composition. Furthermore, incorporation of Mg increased in all the four elevated temperature treatments compared to the control. In line with our results, incorporation of Mg linearly increased with culture temperature in several species of planktonic foraminifera [72], [73] and marine mussel [14]. Calcifiers such as coccolithophores, foraminifera, corals and bivalves have been shown to partition strontium despite their different calcification mechanisms [63], [74], [75]. The Sr/Ca ratio has been shown to have different degrees of responsiveness to environmental variables, temperature, aragonite saturation states and salinity [76], and has been suggested as a potential proxy for calcification rates [77], [78], [79]. Similar to previous findings [77], the Sr/Ca ratio was consistently lower at 29°C than in the control. This could have been partially driven by a higher precipitation rate of minerals [76]. With values nearer to undersaturation, the Sr/Ca ratio was more responsive. It is not yet known how reduction of Ω_A_ affects the Sr/Ca ratio in biominerals, because the direction and magnitude of change are also dependent on taxonomic differences [80]. This complex and non-linear relationship with Ω_A_ requires further investigation.

### Tube Mechanical Properties: Effect of pH, Salinity and Temperature

Results from the nanoindentation showed that at elevated temperature, the tubes were harder and more elastic, regardless of salinity and pH, indicating a recovery from the negative effects caused by decreased pH. The basis of the positive effect on tube hardness by elevated temperature remains unclear. Nevertheless, such beneficial effects might be indirectly related to alterations of seawater carbonate chemistry by changes in temperature, i.e. high temperature increases saturation states. This conclusion is partially supported by the observed positive correlation between the tube hardness and one of the carbonate system variables, CO_3_^2-^. The CO_3_^2-^ concentration (70 µmol kg^−1^) at elevated temperature of 29°C with salinity of 27‰ and pH 7.8 was similar to the CO_3_^2-^ concentration (80 to 82 µmol kg^−1^) found at ambient temperature and reduced salinity or reduced pH (Table 1). Similarly, a study had shown snails in warm (tropical) waters build shells that are stronger providing better protection (enhanced mechanical strength) than those living in cold (temperate) waters [81]. This temperature effects may be potentially important for calcifying organisms experiencing ocean acidification damage in terms of shell hardness reduction as observed previously [82].

### Tube Structure: Effect of pH, Salinity and Temperature

The tubeworm produced normally structured tubes with a distinctive irregularly ordered prismatic (IOP) inner layer and round homogeneous crystal (RHC) structured middle layer (Figure 7) in all three stress levels examined in this study. Additionally, the tubes produced at elevated temperature had a unique accretion of a spherulitic prismatic (SPHP) outer layer on a pair of “ridges” and these tubes appeared less porous and more compact compared to those produced at 23°C. The outermost layer is commonly recognized to be important for resisting corrosion and external mechanical attacks [35], and the presence of “ridge” like structures might serve as a strengthening ultrastructural feature. The mechanical function of various tube ultrastructures should be further investigated using high resolution mapping [31], [83], [84]. Nevertheless, the presence of more advanced oriented and semi-oriented tube ultrastructures [66] and the ability to maintain these features at elevated temperature may indicate considerable biological control exerted by tubeworms to produce normal or even stronger armor in response to unfavorable marine environments such as decreased pH and reduced salinity in coastal waters.

### Potential Tubeworm Population Responses

Except in extreme monsoonal events, the eastern Hong Kong waters are generally 29‰ and 25°C in the wet season [39], which are often milder than those tested levels in the present study. Warming treatment (29°C) in this study seemed to offset some of the negative impacts of reduced salinity and pH, and seemed to benefit tube accretion in terms of composition, structure, and mechanical features. The positive responses to a warming temperature 29°C suggest a potential coping ability of the animal in the field with the near future projected condition. In order to explain why population declines in the field during the summer, future studies should further investigate other environmental parameters associated with seasonality such as food availability [85], and larval source [86].

## Conclusion

Combinations of decreased pH, reduced salinity or elevated temperature did not detrimentally affect the mineral composition, structure or mechanical strength of tubes constructed by *H. elegans*. Moreover, these conditions did not cause any detrimental structural defects that led to a mechanically weaker tube. It seems that elevated temperature counteracts the negative effects of decreased pH and salinity on tube mechanical properties by strengthening the tube structure. Our results from the analyses of tube mineralogy, ultrastructure and mechanical properties showed that predicted coastal warming may not hinder *H. elegans* ability to build normal tubes even in the face of projected near-future decreases in pH or salinity. The results also suggests that intensity of hard structures built by *H. elegans*, i.e. biofouling, will not be reduced and may in fact become slightly stronger under elevated temperature, decreased pH and reduced salinity. However, our understanding of biofouling in changing coastal water environments is poor and also needs further study. Population survival and fitness not only depend on production of the calcareous tube but also on larval recruitment, survival, growth, reproduction and energy balance [87]. Therefore, we need a holistic approach to experimental design to study the effect of environmental variables on *H. elegans* that encompasses aspects of their development, physiology and biomineralization processes to predict population dynamics in a changing coastal climate.

## Supporting Information

Table S1 **Results of 3-way analysis of variance (ANOVA) showing the effect of temperature (23°C and 29°C), salinity (27 and 34 ‰) and pH (8.1 and 7.8) on amorphous calcium carbonate (CaCO_3_) (ACC) content and calcite to aragonite ratio in the calcareous tube of** ***Hydroides elegans*** **.** Significant effects (*p*<0.05) are indicated in bold. Data for ACC content were square root transformed whereas calcite/aragonite ratio data were log transformed to improve homogeneity of variance.
(DOCX)

Table S2 **Results of 3-way analysis of variance (ANOVA) showing the effect of temperature (23°C and 29°C), salinity (27 and 34 ‰) and pH (8.1 and 7.8) on magnesium to calcium (Mg/Ca) and strontium to calcium (Sr/Ca) ratios in the calcareous tube of** ***Hydroides elegans*** **.** Significant effects (*p*<0.05) are indicated in bold. Data for Mg/Ca were log transformed to improve homogeneity of variance. *As Mg/Ca ratio did not fulfill requirement of variance homogeneity, the critical p-value should be adjusted to a more conservative value of p<0.01. Therefore, the bold values should be interpreted with caution.
(DOCX)

Table S3 **Results of 3-way analysis of variance (ANOVA) showing the effect of temperature (23°C and 29°C), salinity (27 and 34 ‰) and pH (8.1 and 7.8) on hardness (H) and Young’s modulus of elasticity (E) of the calcareous tube in** ***Hydroides elegans*** **.** Significant effects (*p*<0.05) are indicated in bold. Data were square root transformed to improve homogeneity of variance.
(DOCX)
