# Supplementary material for: Temperature Dependent Effects of Elevated CO2 on Shell Composition and Mechanical Properties of Hydroides elegans: Insights from a Multiple Stressor Experiment
Source: PLoS One. 2013 Nov 12;8(11):e78945. doi: 10.1371/journal.pone.0078945 (PMC3827122; doi:10.1371/journal.pone.0078945)
Supplement: Table S1 — Results of 3-way analysis of variance (ANOVA) showing the effect of temperature (23°C and 29°C), salinity (27 and 34 ‰) and pH (8.1 and 7.8) on amorphous calcium carbonate (CaCO3) (ACC) content and calcite to aragonite ratio in the calcareous tube of Hydroides elegans . Significant effects (p<0.05) are indicated in bold. Data for ACC content were square root transformed whereas calcite/aragonite ratio data were log transformed to improve homogeneity of variance. (DOCX) [file pone.0078945.s001.docx]

**Table S1**

Results of 3-way analysis of variance (ANOVA) showing the effect of temperature (23^o^C and 29^o^C), salinity (27 and 34 ‰) and pH (8.1 and 7.8) on amorphous calcium carbonate (CaCO_3_) (ACC) content and calcite to aragonite ratio in the calcareous tube of *Hydroides elegans*. Significant effects (*p* < 0.05) are indicated in bold. Data for ACC content were square root transformed whereas calcite/aragonite ratio data were log transformed to improve homogeneity of variance.

| Factor |  | **Amorphous CaCO_3_** | | | | | **Calcite/aragonite** | | | |
| --- | --- | --- | --- | --- | --- | --- | --- | --- | --- | --- |
|  | df | MS | F | p |  | MS | | F | p |  |
|  |  |  |  |  |  |  | |  |  |  |
| pH | 1 | 5.222 | **20.865** | <0.001 |  | 0.919 | | **6.144** | 0.021 |  |
| Salinity | 1 | 0.399 | 1.593 | 0.219 |  | 0.016 | | 0.107 | 0.747 |  |
| Temperature | 1 | 10.472 | **41.841** | <0.001 |  | 0.681 | | **4.557** | 0.043 |  |
| pH × Salinity | 1 | 0.889 | 3.553 | 0.072 |  | 0.242 | | 1.615 | 0.216 |  |
| pH × Temperature | 1 | 0.306 | 1.223 | 0.280 |  | 0.477 | | 3.190 | 0.087 |  |
| Salinity × Temperature | 1 | 0.060 | 0.239 | 0.630 |  | <0.001 | | <0.001 | 0.993 |  |
| pH × Salinity × Temperature | 1 | <0.001 | <0.001 | 0.990 |  | 3.7 | | 2.148 | 0.156 |  |
| Error | 24 | 0.250 |  |  |  | 4.2 | |  |  |  |
