# Supplementary material for: Temperature Dependent Effects of Elevated CO2 on Shell Composition and Mechanical Properties of Hydroides elegans: Insights from a Multiple Stressor Experiment
Source: PLoS One. 2013 Nov 12;8(11):e78945. doi: 10.1371/journal.pone.0078945 (PMC3827122; doi:10.1371/journal.pone.0078945)
Supplement: Table S2 — Results of 3-way analysis of variance (ANOVA) showing the effect of temperature (23°C and 29°C), salinity (27 and 34 ‰) and pH (8.1 and 7.8) on magnesium to calcium (Mg/Ca) and strontium to calcium (Sr/Ca) ratios in the calcareous tube of Hydroides elegans . Significant effects (p<0.05) are indicated in bold. Data for Mg/Ca were log transformed to improve homogeneity of variance. *As Mg/Ca ratio did not fulfill requirement of variance homogeneity, the critical p-value should be adjusted to a more conservative value of p<0.01. Therefore, the bold values should be interpreted with caution. (DOCX) [file pone.0078945.s002.docx]

**Table S2**

Results of 3-way analysis of variance (ANOVA) showing the effect of temperature (23^o^C and 29^o^C), salinity (27 and 34 ‰) and pH (8.1 and 7.8) on magnesium to calcium (Mg/Ca) and strontium to calcium (Sr/Ca) ratios in the calcareous tube of *Hydroides elegans*. Significant effects (*p* < 0.05) are indicated in bold. Data for Mg/Ca were log transformed to improve homogeneity of variance. *As Mg/Ca ratio did not fulfill requirement of variance homogeneity, the critical p-value should be adjusted to a more conservative value of p < 0.01. Therefore, the bold values should be interpreted with caution.

| Factor |  | **Mg/Ca** | | | | | **Sr/Ca** | | | |
| --- | --- | --- | --- | --- | --- | --- | --- | --- | --- | --- |
|  | df | MS | F | p |  | MS | | F | p |  |
|  |  |  |  |  |  |  | |  |  |  |
| pH | 1 | 1075.420 | **5.393** | 0.029 |  | 0.445 | | 1.019 | 0.323 |  |
| Salinity | 1 | 222.541 | 1.116 | 0.301 |  | 0.008 | | 0.017 | 0.896 |  |
| Temperature | 1 | 1055.588 | **5.293** | 0.030 |  | 2.256 | | **5.164** | 0.032 |  |
| pH × Salinity | 1 | 148.411 | 0.744 | 0.397 |  | 0.140 | | 0.320 | 0.577 |  |
| pH × Temperature | 1 | 341.827 | 1.714 | 0.203 |  | 0.491 | | 1.123 | 0.300 |  |
| Salinity × Temperature | 1 | 41.495 | 0.208 | 0.652 |  | 0.778 | | 1.782 | 0.194 |  |
| pH × Salinity × Temperature | 1 | 163.696 | 0.821 | 0.374 |  | 2.263 | | **5.180** | 0.032 |  |
| Error | 24 | 199.419 |  |  |  | 0.437 | |  |  |  |
