# Supplementary material for: Temperature Dependent Effects of Elevated CO2 on Shell Composition and Mechanical Properties of Hydroides elegans: Insights from a Multiple Stressor Experiment
Source: PLoS One. 2013 Nov 12;8(11):e78945. doi: 10.1371/journal.pone.0078945 (PMC3827122; doi:10.1371/journal.pone.0078945)
Supplement: Table S3 — Results of 3-way analysis of variance (ANOVA) showing the effect of temperature (23°C and 29°C), salinity (27 and 34 ‰) and pH (8.1 and 7.8) on hardness (H) and Young’s modulus of elasticity (E) of the calcareous tube in Hydroides elegans . Significant effects (p<0.05) are indicated in bold. Data were square root transformed to improve homogeneity of variance. (DOCX) [file pone.0078945.s003.docx]

**Table S3**

Results of 3-way analysis of variance (ANOVA) showing the effect of temperature (23^o^C and 29^o^C), salinity (27 and 34 ‰) and pH (8.1 and 7.8) on hardness (H) and Young's modulus of elasticity (E) of the calcareous tube in *Hydroides elegans*. Significant effects (*p* < 0.05) are indicated in bold. Data were square root transformed to improve homogeneity of variance.

| Factor |  | **Hardness** | | | | | ***Elasticity*** | | | |
| --- | --- | --- | --- | --- | --- | --- | --- | --- | --- | --- |
|  | df | MS | F | p |  | MS | | F | p |  |
|  |  |  |  |  |  |  | |  |  |  |
| pH | 1 | 2.923 | 1.937 | 0.177 |  | 440.231 | | 1.329 | 0.260 |  |
| Salinity | 1 | 4.215 | 2.794 | 0.108 |  | 626.262 | | 1.891 | 0.182 |  |
| Temperature | 1 | 8.444 | **5.597** | 0.026 |  | 1881.850 | | **5.683** | 0.025 |  |
| pH × Salinity | 1 | 0.297 | 0.197 | 0.661 |  | 5.875 | | 0.018 | 0.895 |  |
| pH × Temperature | 1 | 1.454 | 0.963 | 0.336 |  | 256.348 | | 0.774 | 0.388 |  |
| Salinity × Temperature | 1 | 2.554 | 1.693 | 0.206 |  | 513.943 | | 1.552 | 0.225 |  |
| pH × Salinity × Temperature | 1 | 0.015 | 0.01 | 0.922 |  | 47.554 | | 0.144 | 0.708 |  |
| Error | 24 | 1.509 |  |  |  | 331.164 | |  |  |  |
